# Space-time dispersion of dengue occurrence in epidemic and non-epidemic years in a municipality in the metropolitan region of Belo Horizonte, MG, 2011 to 2017

**DOI:** 10.1590/1980-549720240023

**Published:** 2024-06-14

**Authors:** Selma Costa de Sousa, Juliana Maria Trindade Bezerra, Diogo Tavares Cardoso, Fabrício Thomaz de Oliveira Ker, Giovanna Rotondo de Araújo, Vagner Braga Nunes Coelho, David Soeiro Barbosa

**Affiliations:** I Universidade Federal de Minas Gerais, Institute of Biological Sciences, Department of Parasitology, Postgraduate Program in Parasitologia – Belo Horizonte (MG), Brazil.; II Universidade Estadual do Maranhão, Campus de Lago da Pedra – Lago da Pedra (MA), Brazil.; III Universidade Estadual do Maranhão, Postgraduate Program in Ciência Animal – São Luís (MA), Brazil.; IV Universidade Federal de Minas Gerais, Institute of Geosciences, Department of Cartography, Postgraduate Program in Environmental Systems Analysis and Modeling – Belo Horizonte (MG), Brazil.

**Keywords:** Dengue, Spatial analysis, Epidemiology, Epidemics, Dengue, Análise espacial, Epidemiologia, Epidemias

## Abstract

**Objective:**

To analyze the transmission dynamics of dengue, a public health problem in Brazil and the Metropolitan Region of Belo Horizonte (MRBH).

**Methods:**

The spatiotemporal evolution of the occurrence of dengue in the municipality of Contagem, state of Minas Gerais, a region with high arbovirus transmission, was analyzed. Furthermore, epidemic and non-epidemic periods were analyzed, based on probable cases of dengue. This is an ecological study that used the Notifiable Diseases Information System (SINAN) national database. The analyses were carried out considering the period from epidemiological week (EW) 40 of 2011 to 39 of 2017. Spatial analysis tools (crude and smoothed incidence rate, directional distribution ellipse, global Moran index and local Moran index, and spatial scanning time with definition of epidemiological risk) were used.

**Results:**

The 2012 to 2013 and 2015 to 2016 epidemic cycles presented high incidence rates. The disease was concentrated in more urbanized areas, with a small increase in cases throughout the municipality. Seven statistically significant local clusters and areas with a high rate of cases and accentuated transmission in epidemic cycles were observed throughout the municipality. Spatial autocorrelation of the incidence rate was observed in all periods.

**Conclusion:**

The results of the present study highlight a significant and heterogeneous increase in dengue notifications in Contagem over the years, revealing distinct spatial patterns during epidemic and non-epidemic periods. Geoprocessing analysis identified high-risk areas, a piece of knowledge that can optimize the allocation of resources in the prevention and treatment of the disease for that municipality.

## RESUMO

**Objetivo:**

Analisar a dinâmica de transmissão da dengue, problema de saúde pública no Brasil e Região Metropolitana de Belo Horizonte (RMBH).

**Métodos:**

Foi analisada a evolução espaçotemporal da ocorrência de dengue no município de Contagem, estado de Minas Gerais, região de alta transmissão da arbovirose. Ainda, foram analisados períodos epidêmicos e não epidêmicos, com base em casos prováveis de dengue. Trata-se de um estudo ecológico que utilizou o banco de dados nacional do Sistema de Informação de Agravos de Notificação (SINAN). As análises foram realizadas considerando-se o período compreendido da semana epidemiológica 40 de 2011 a 39 de 2017. Ferramentas de análise espacial (taxa de incidência bruta e suavizada, elipse de distribuição direcional, índice de Moran global e índice de Moran local, e varredura espaçotemporal com definição do risco epidemiológico) foram utilizadas.

**Resultados:**

Os ciclos epidêmicos 2012 a 2013 e 2015 a 2016 apresentaram altas taxas de incidência. A doença concentrou-se em áreas mais urbanizadas, com pequena expansão de casos por todo o município. Foram observados sete aglomerados locais estatisticamente significativos e áreas com alta taxa de casos e transmissão acentuada em ciclos epidêmicos em todo o município. A autocorrelação espacial da taxa de incidência foi observada em todos os períodos.

**Conclusão:**

Os resultados do presente estudo destacam um aumento significativo e heterogêneo nas notificações de dengue em Contagem ao longo dos anos, revelando padrões espaciais distintos durante períodos epidêmicos e não epidêmicos. A análise de geoprocessamento identificou áreas de alto risco, conhecimento este que pode otimizar a alocação de recursos na prevenção e tratamento da doença para o referido município.

## INTRODUCTION

Dengue is a notifiable disease in Brazil and all suspected or confirmed cases need to be registered in the Notifiable Diseases Information System (*Sistema de Informação de Agravos de Notificação* — SINAN)^1^. The occurrence of arbovirus epidemics in the country has become a matter of increasing concern for the public health authorities, due to the difficulties faced in relation to these diseases^2-4^. Brazil notified 4,500,111 probable cases of dengue between 2016 and 2020, with 1,486,970 (33.0%) in 2016; 241,551 (5.4%) in 2017; 267.798 (6.0%) in 2018; 1,551,452 (34.5%) in 2019; and 952,340 (21.2%) in 2020^5^. In 2021, between epidemiological weeks (EW) 1 and 40 of 2022, 1,362,125 probable cases of dengue were notified, an increase of 184.6% in the registered cases for the same period observed in the previous year^6^.

Minas Gerais, a state of Brazil’s Southeast Region, had its first epidemic in 1995 in the municipality of Nanuque, in the northeastern area of the state, with the isolation of DENV-2. The disease has since spread, with outbreaks that have accompanied the major epidemics reported in the country. Since 2011, the four serotypes have been identified in co-circulation in Minas Gerais, with the serotype DENV-1 circulating predominantly until 2017 and DENV-2 predominating since 2018. In 2019, the four serotypes were identified, and in 2020, DENV-1 was identified in three municipalities, while DENV-2 was identified in other 27 municipalities^7^. In 2019, 474,000 probable cases and 188 deaths were notified, with a decrease to 84,636 probable cases and 15 dengue deaths in 2020^8^. As in other regions of Brazil, lack of basic sanitation, low per capita gross domestic product (GDP) and high population density have contributed to the spread of dengue in municipalities in Minas Gerais^9-11^.

In the Metropolitan Region of Belo Horizonte (MRBH), which is composed of 14 municipalities, 264,162 probable cases of dengue were notified from 2019 to August 2021, of which 250,990 (95.0%) occurred in 2019, 10,652 (4.0%) in 2020 and 2,520 (1.0%) in 2021. Contagem accounted for 15% of the total cases^5^. In Contagem, the disease is endemic and epidemic peaks occur every three to four years. Over the last ten years, epidemics occurred in 2012, 2016 and 2019. From 2019 to January 2022, there were 56,655 cases of the disease in the municipality, and most of the notifications were in 2019:52,144 (92.0%)^5^.

The use of geoprocessing analysis on dengue cases^12,13^, focusing on space-time analyses of the disease in a given region, locality or country, allows healthcare managers to generate hypotheses that can explain the real occurrence rates of the disease. These techniques make it possible for ecological studies to incorporate a variety of factors for determining disease occurrence patterns, by including the effects of the specific characteristics of each social space. This supports healthcare professionals in the challenging task of combating the disease through planned prevention and coping actions, such as predicting the number of dengue cases in other places or future times^14-16^.

To anticipate the spread of dengue fever in the city of Contagem, local public authorities can use case spatialization tools. These analyses reveal patterns of disease spread in different periods, allowing a more in-depth understanding of the places with the highest incidence. By identifying high-risk areas during both epidemic and non-epidemic periods, the community can adopt more targeted preventive measures. Recognizing hot areas for disease transmission in epidemic years enables a more agile and effective response, contributing to a more efficient distribution of resources destined for the prevention and treatment of dengue in the city. This proactive approach based on spatial data is crucial to strengthening society’s ability to deal with the challenges associated with the spread of the disease.

Therefore, the aim of the present study was to analyze the temporal and spatial evolution of dengue incidence in Contagem, state of Minas Gerais, in epidemic cycles (2012–2013 and 2015–2016) and non-epidemic cycles (2011–2012, 2013–2014, 2014–2015, and 2016–2017).

## METHODS

### Study area

**Figure 1:**
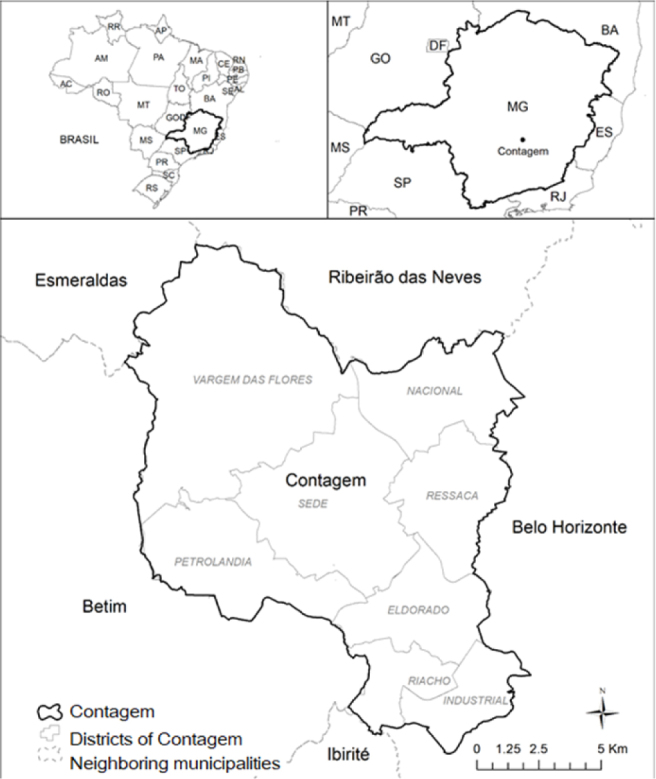
Municipality of Contagem (MG), Brazil, showing the eight districts.

The municipality of Contagem plays a central role in the urban economic activities of the MRBH. It has an area of 195,268 km^2^, density of 3,375.21 inhabitants/km^2^ in 2010, an average altitude of 858.00 meters and high-altitude tropical climate. Its estimated population in 2020 was 668,949 inhabitants^17,18^, and it is divided into eight districts (Figure 1): Industrial (30 neighborhoods), Ressaca (48 neighborhoods), Nacional (39 neighborhoods), Petrolândia (15 neighborhoods), Centro (65 neighborhoods), Vargem das Flores (19 neighborhoods) and Eldorado, which in 2016 was subdivided into Eldorado (22 neighborhoods) and Riacho (17 neighborhoods)^19^.

### Study design and data sources

This is an ecological study in which aggregated secondary data relating to notified probable dengue cases among residents of the municipality of Contagem between 2011 and 2017 were used. SINAN databases last updated on October 2, 2019, were used, and population data for 2010, the last available national census, were obtained from the Brazilian Institute of Geography and Statistics (*Instituto Brasileiro de Geografia e Estatística* — IBGE)^17^.

### Geoprocessing and spatial analysis

The databases obtained from SINAN were organized and unified according to cycle (between EW40 of one year and 39 of the following year), using the reference point of the week of symptom onset. The Microsoft Office Excel 2013 software (Washington, USA) was used to organize the data. Probable dengue cases were georeferenced from the patients’ addresses registered in the database. Each coordinate corresponded to the notification of a new probable case of dengue in each EW. Google Maps (USA) was used to obtain coordinates that were as close as possible to the address, with the street and block as references. After establishing these geographical coordinates, a shape file was built as a series of points in the QGIS Development Team 2019 application (Bucharest, Romania).

All dengue cases were selected according to epidemiological cycles, considering the week of onset of symptoms (SEM) in all cases, starting in SE 40 in 2011 and ending in SE 39 in 2017. Thus, 56,559 probable cases were available for spatial analysis.

#### Crude and smoothed incidence rates

The crude incidence rates of probable dengue cases were calculated by aggregating data from the six epidemiological cycles. The total number of cases formed the numerator, and the population data for each area formed the denominator, using data from the 2010 IBGE census^17^ multiplied by 100,000 inhabitants. Crude incidence rates were calculated for each of the geographical units (census tracts) and for each cycle, using Bayesian spatial smoothing, in the GeoDa software, version 1.10 (Arizona, USA).

The Local Empirical Bayesian method was used to correct random fluctuations in dengue incidence rates occurring in areas with few cases and small populations. Geographic neighbors were considered to estimate the risk of the disease occurring in each area analyzed.

The smoothed indicators obtained were represented by means of equal class intervals. The data was visualized using choropleth maps, using different colors for each interval used.

#### Directional distribution ellipse

The directional distribution ellipse (or standard deviation ellipse) technique helps identify the distribution of points (events) by drawing an ellipse. It is determined through four parameters: angle of rotation, dispersion along the major axis, dispersion along the minor axis and mean center (or spatial center). The major axis defines the direction of maximum dispersion of the distribution, while the minor axis is perpendicular to the previous axis and defines the minimum dispersion^20^.

The directional distribution ellipse was calculated in three steps. First, the average center of the distribution was evaluated. Next, the orientation of the axes that defined the ellipse were determined through the least-squares method, which aimed to minimize the sum of variances. Lastly, the standard deviations of the latitude and longitude axes were calculated^20^. The directional distribution was applied using the QGIS software, version 3.10 (Bucharest, Romania).

An advantage of the standard deviation ellipse is that through the ellipse it is possible to know the orientation of dengue cases in the municipality of Contagem.

#### Global Moran index and local Moran index

Spatial dependence patterns were investigated by calculating the global Moran index (GMI) (I value) and the local Moran index (LMI)^21,22^. To obtain these indexes, a first-order neighborhood matrix (Queen) was created to ascertain the dependency relationship among the areas, considering neighbors to be areas that bordered each other^22^.

The local Moran index is a statistical tool that tests local autocorrelation and detects spatial objects with an influence on I. While the global Moran index informs the level of spatial interdependence between all polygons under study, the local Moran index evaluates covariance between a given polygon and a neighborhood defined as a function of a distance.

To identify different patterns in the analysis of the units that presented locations where spatial dependence was more pronounced, the local Moran index (LMI) needed to be used. This indicator was used to identify spatial clusters, which were determined for successive three-year periods. The LMI presents a normalized value (values of attributes subtracted from its mean and divided by the standard deviation) for each area, which enables the identification of clusters of areas with significant patterns for spatial association. The LMI analysis decomposes the value of the GMI, thus reflecting the value of the analysis unit and whether it is associated with its neighbors and shows the presence or absence of positive values^22^.

The GMI was used to evaluate the overall clustering. GMI values range from -1, which means negative spatial dispersion or autocorrelation, to +1, indicating positive spatial grouping or autocorrelation^23^. Since global spatial analysis produces only one value summarizing the entire study area, LMI was also used to determine cluster dependence between census tracts of the municipality. Thus, each census tract was classified according to its position within the quadrants of Moran’s scatter plot as follows: Q1 (+/+) positive spatial autocorrelation and positive values in neighboring location; Q2 (-/-) negative spatial autocorrelation and negative values in neighboring location; Q3 (+/-) positive spatial autocorrelation and negative values in neighboring location; and Q4 (-/+) negative spatial autocorrelation and positive values in neighboring location^24^.

GMI and LMI were calculated using the GeoDa software, version 1.10 (Arizona, USA), and maps were constructed using the QGIS software, version 3.10 (Bucharest, Romania).

### Space-time scanning and definition of epidemiological risk

Space-time scanning is a technique that evaluates incidence rates through a circle that moves in space, changing the radius and position of its center through a scan of the space where it is being studied, locating a circle in the place where there is a high incidence rate, inside, and very low, outside this^20^. Population information according to census tract was taken from the 2010 IBGE census^17^.

Spatial clusters were identified using Poisson’s discrete model. To calculate scanning statistics, the following configurations were used: no occurrence of geographical overlap of the clusters; clusters of maximum size equal to 50% of the exposed population; maximum size of the time clusters equal to 50% of the study period; and time accuracy standardized in one year, with circular sets and 999 repetitions. We chose to use the default mode settings in the analysis so that the choice is not arbitrary. Thus, 50% of the population is considered as the maximum size of the spatial and temporal cluster. With this, SaTScan will then evaluate very small and very large clusters, and everything in between. This model considered the space and time in which the cases occurred^22,23^. To identify spatial and space-time clusters, scanning statistics were applied using the SaTScan software, version 9.4.4 (Boston, USA)^25^.

## RESULTS

### Risk mapping

**Figure 2:**
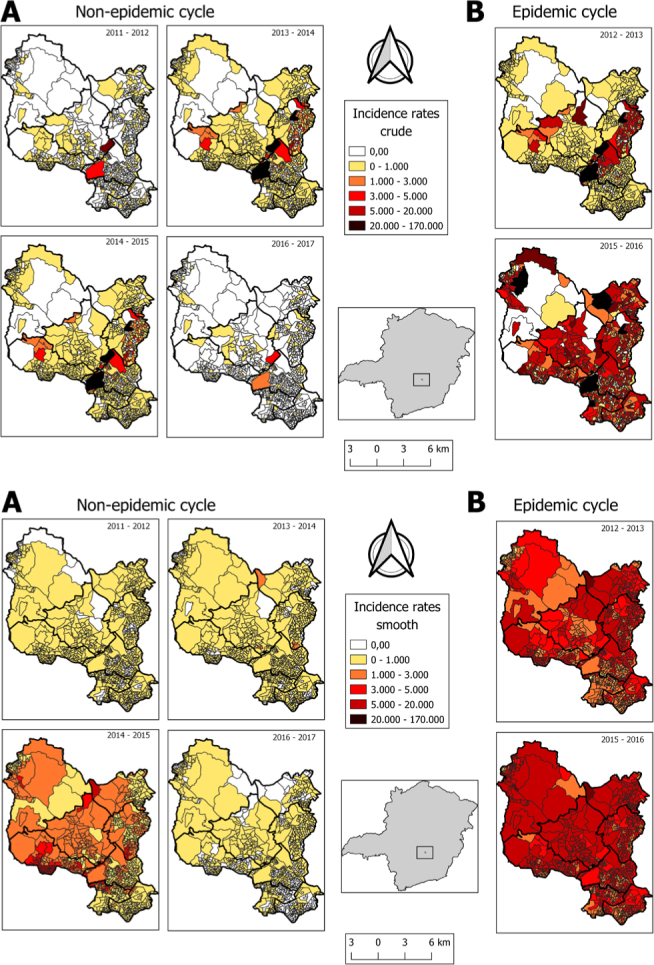
Incidence rate for dengue in the municipality of Contagem (MG), described in six epidemiological cycles*. (A) Accumulated gross incidence rate (B) Smoothed incidence rate.

During the epidemic periods 2012–2013 and 2015–2016, the highest rates of dengue incidence for the entire municipality were recorded. The greatest numbers of census tracts, with rates that reached 170,000 cases per 100,000 inhabitants, were recorded during these periods. Regarding non-epidemic periods, high incidence was observed in the 2014–2015 cycle before the explosion of the largest dengue epidemic in the 2015–2016 cycle. In the other non-epidemic periods (2011–2012, 2013–2014, and 2016–2017), the incidence rates ranged from 0 to 1,000 cases per 100,000 inhabitants in most census tracts (Figure 2 A and B).

**Figure 3:**
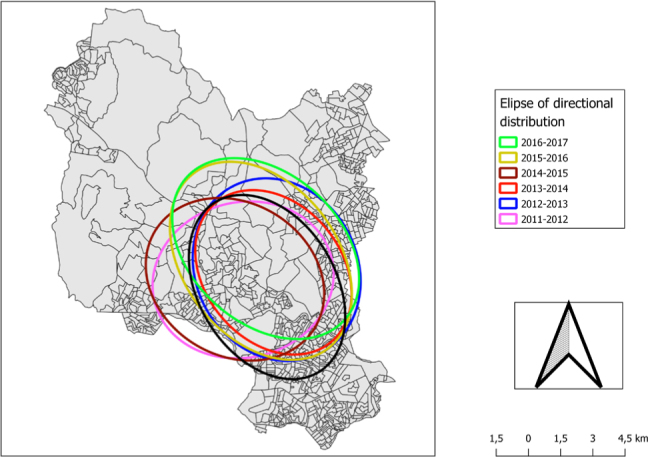
Directional distribution of dengue probable cases in the municipality of Contagem (MG) in six epidemiological cycles in the period of 2011–2017.

Concentrations were observed in more urbanized areas (Centro, Ressaca, Eldorado and Riacho districts), with a slight expansion of dengue cases influenced by epidemics westwards to Petrolândia district (2011–2012 and 2014–2015) and northwards to the Vargem das Flores rural area (2015–2016 and 2016–2017) (Figure 3).

### Global and local Moran indexes

**Figure 4:**
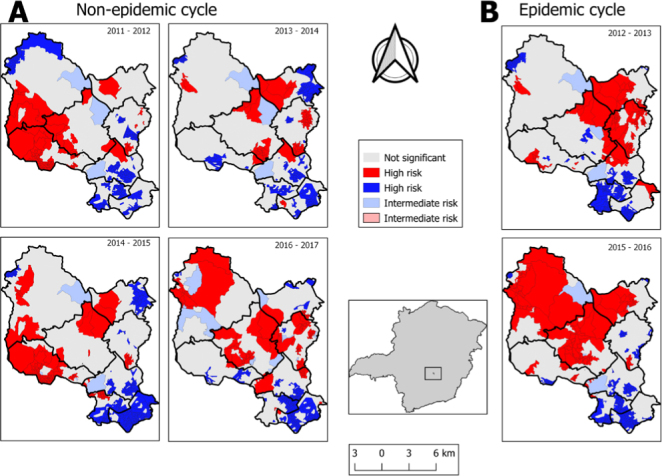
Moran maps of the average dengue incidence rate by census tracts in the non-epidemic (A) and epidemic (B) cycles and Moran’s Scatter Diagram.

Significant spatial autocorrelations were observed over the study period. In the non-epidemic cycles, most census tracts presented spatial clusters for dengue. Vargem das Flores was the region with the lowest risk, except in the 2016–2017 period. It was observed that the four non-epidemic cycles had similar clusters, with high-high and low-low spatial patterns, and this was repeated in census tracts in the four maps referring to the cycles 2011–2012, 2013–2014, 2014–2015 and 2016–2017 (Figure 4A). The non-epidemic cycles showed high spatial dependence, with the following GMI values: 2011–2012 (I=0.752); 2013–2014 (I=0.639); 2014–2015 (I=0.793) and 2016–2017 (I=0.523) (Figure 4A). In the epidemic cycles, there were also high GMI spatial correlations: 2012–2013 (I=0.587) and 2015–2016 (I=0.523) (Figure 4B).

### Space-time scanning analysis

Three statistically significant local clusters (1, 2, and 3) were detected for the epidemic period 2012–2013 and four clusters (1, 2, 3, and 4) for the epidemic period 2015–2016. For the non-epidemic periods, five clusters were detected for the period 2011–2012, five for the period 2013–2014, three for the period 2014–2015 and two for the period 2016–2017. The foci of the clusters were on different locations and had time intervals of different sizes.

**Figure 5:**
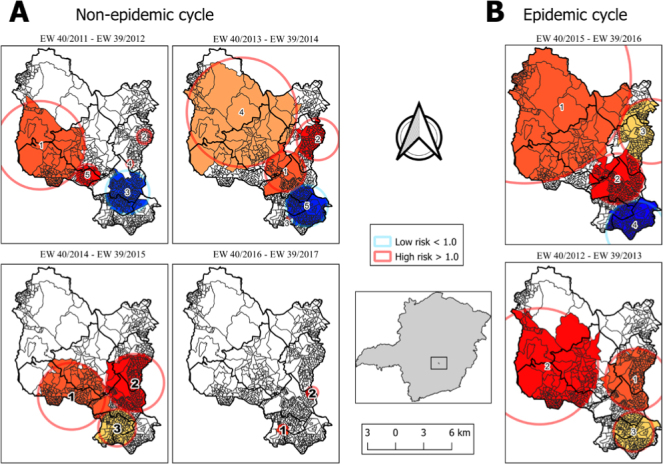
Analysis of the spatiotemporal permutation method of probable cases of dengue in the municipality of Contagem (MG) in six epidemiological cycles in the period of 2011–2017.

Risks of dengue occurrence were detected in all intervals and clusters with high and very high risks. For example, in the non-epidemic period of 2013–2014, cluster 3 presented a relative risk (RR) of 117,17; and in the period 2016–2017, cluster 2 presented a RR of 112.25 (Figure 5).

## DISCUSSION

In the municipality of Contagem, state of Minas Gerais, dengue has presented worrying epidemic episodes over recent years. This study analyzed the space-time evolution of dengue incidence over a period of non-epidemic cycles in 2011–2012, 2013–2014 and 2016–2017; and epidemic cycles in 2012–2013 and 2015–2016. Spatial and temporal analyses can describe whether certain phenomena occur randomly or whether they are clustered in specific areas. In the present study, census tracts were chosen as data aggregators because these were the smallest geographical units available from IBGE^17^ and because this allowed spatial evaluation of the areas where the highest levels of transmission of the disease were concentrated.

During the two major dengue epidemics, in the periods 2012–2013 and 2015–2016, the incidence of dengue was high in almost the entire municipality. Among the non-epidemic cycles, the incidence was high in relation to other non-epidemic periods only the period 2014–2015. In other studies, conducted in the city of São José do Rio Preto (SP), during an epidemic that occurred in the first half of 1995, in which census tracts were evaluated, it was observed that the highest incidences of the disease were in areas with worse living conditions^14^; in areas in which socioeconomic variables were associated with differences in disease classifications, as per the same study; in situations in which the disease occurred independently in relation to socioeconomic profiles in Rio de Janeiro in 2022 and in São José do Rio Preto, state of São Paulo, between September 1990 and August 2002^12,26^; or in endemic periods when the incidence of dengue was scattered, while in epidemic periods the incidence had a uniform distribution within the municipality^27^.

In the present study, directional analysis, representation of means and analysis of arbovirus dispersion according to distance from points showed that the displacement of ellipses was only slight within the same region of the municipality, in all epidemiological cycles, thus characterizing strong regularity over the period analyzed period. The region highlighted was the Centro district, a region that has a predominantly middle-class and lower middle-class population. The less central neighborhoods of the district have shown intense population growth over the years. Other studies, in Porto Alegre, from January to July 2002, and Paraíba, from 2011 to 2014, using the same technique, have shown similar results^28,29^. However, a further study in greater depth can be suggested to highlight the entomological and climatological aspects of the disease, to seek more evidence regarding dengue transmission in this region.

A significant spatial autocorrelation of dengue incidence in the municipality was observed in all study cycles, with high risk of arbovirus transmission. Moran’s indicators obtained in this study showed positive values that were indicative of positive spatial autocorrelations. There was a strong relationship between census tracts and an even greater one among nearest neighboring census tracts. Moran’s index showed significant positive spatial correlations for all six epidemiological cycles (both non-epidemic and epidemic), thus corroborating the findings from studies conducted in Bayeux (PB)^29^, Belo Horizonte (MG)^30^, Ceará^31^, Rio de Janeiro (RJ)^12^, Piracicaba (SP)^32^ and Brazil^33^. For this reason, the risk assessment regarding the living conditions of residents in the different census tracts of the municipality can be invaluable information for future studies.

The spatiotemporal scanning analysis made it possible to identify clusters at high risk of dengue in different regions of the municipality. These clusters persisted in all epidemic and non-epidemic cycles studied, in some regions of the municipality. The persistence of the disease can be explained by the simultaneous circulation of different serotypes of dengue (1 and 4)^33^ and may also have been due to socioeconomic and environmental factors^12,26,34-36^.

For the present study, official data from SINAN were used. However, it is known that these data are subject to limitations. Because asymptomatic people are often unaware of their conditions, they do not go to healthcare units and so their conditions are not notified. It is also observed that the data submitted in notifications are sometimes incomplete, with blank fields, incorrect addresses, illegible writing, repetitiveness, and inconsistency of information, such as non-closure of cases. This lack of information makes it difficult for analyses to be more accurate and comprehensive. Furthermore, in the analyses demonstrated here, the patients’ home addresses were used. For this reason, these addresses were the probable sites of infection, even though home addresses are often not the true location where infection was transmitted. However, our study provides an evaluation of the space-time evolution of dengue incidence in Contagem, Minas Gerais, and describes the epidemiology of the disease over a period of six epidemiological cycles, based on probable cases. Spatial statistics tools were used to identify the incidence of dengue and risk areas for occurrences of the disease in the municipality, through the use of time series data.

The findings from the present study demonstrated that dengue notifications in the municipality of Contagem have significantly and heterogeneously increased over the years. The use of geoprocessing studies showed patterns of dissemination of the disease in epidemic and non-epidemic periods. Spatial dependence was observed throughout the period analyzed, with the characterization of epidemic and non-epidemic periods. During the two epidemic cycles, which affected almost the entire municipality, the incidence of dengue was high. It was found that the displacement of ellipses was discrete in the same region of the municipality in all epidemiological cycles, and there was also the identification of high risk for dengue in all epidemiological cycles in the epidemic and non-epidemic periods. In the epidemic years, hot areas for transmission of the disease were detected. This study contributes to a better understanding of the disease patterns in epidemic and non-epidemic years and to a more efficient distribution of resources for prevention and treatment of the disease in municipalities and metropolitan regions of similar contexts.
